# Subcutaneous birch pollen allergen immunotherapy with a depigmented polymerized extract shows only sustained and long‐term efficacy in a subgroup of monosensitized adults and adolescents with allergic rhinitis

**DOI:** 10.1002/clt2.12185

**Published:** 2022-10-05

**Authors:** Natalija Novak, Margitta Worm, Petra Staubach, Marek Jutel, Angelika Sager, Oliver Pfaar

**Affiliations:** ^1^ Department of Dermatology and Allergology University of Bonn Medical Center Bonn Germany; ^2^ Department of Dermatology Allergy and Venerology Division of Allergy and Immunology, Charité Universitätsmedizin Berlin Germany; ^3^ Department of Dermatology University Medical Center of the Johannes Gutenberg University Mainz Mainz Germany; ^4^ Department of Clinical Immunology Wroclaw Medical University Wroclaw Poland; ^5^ ALL‐MED Medical Research Institute Wroclaw Poland; ^6^ LETI Pharma GmbH Witten Germany; ^7^ Department of Otorhinolaryngology Head and Neck Surgery Section of Rhinology and Allergy University Hospital Marburg Philipps‐Universität Marburg Marburg Germany

**Keywords:** allergic rhinitis, birch pollen, long‐term, phase III, post‐treatment, subcutaneous allergen immunotherapy

## Abstract

**Background:**

Allergen immunotherapy (AIT) is an approved treatment for seasonal respiratory allergic diseases. A depigmented polymerized birch pollen extract for subcutaneous allergen immunotherapy (SCIT) has been demonstrated to be efficacious and safe in patients allergic to birch pollen and its homologous group.

**Objective:**

To determine whether SCIT with a birch pollen formulation (5000 depigmented polymerized (DPP) units/mL) shows sustained and long‐term efficacy in adults and adolescents with birch‐pollen induced allergic rhinitis with or without intermittent asthma.

**Methods:**

A multicentre (*n* = 66), double‐blind, placebo‐controlled Phase III clinical trial was performed in the Czech Republic, Finland, Germany, Latvia, Lithuania, Poland and Russia. Participants were randomized 2:1 to active treatment (birch 5000 DPP/ml) or placebo for three years of SCIT and followed up for two treatment‐free years. The primary efficacy endpoint was the EAACI's combined symptom and medication score for rhinoconjunctivitis (CSMS_EAACI_).

**Results:**

A total of 973 participants were screened and 649 were randomized (active treatment: *n* = 434; placebo: *n* = 215). The intention‐to‐treat analysis of the CSMS_EAACI_ in the overall study population did not demonstrate statistically significant differences in years 1, 2 and 3. In a post‐hoc analysis, among the subgroup of patients monosensitized to birch pollen allergen only (*n* = 200), we observed a statistically significant difference (active treatment vs. placebo) in the CSMS_EAACI_ in year 2, 3 and 5. The AIT's safety profile was good.

**Conclusions:**

SCIT with a depigmented polymerized birch pollen extract was safe. Sustained and long‐term efficacy in years 2, 3 and 5 in monosensitized patients, but not in polysensitized patients was demonstrated.

(EudraCT 2012‐000414‐11)

## 1 INTRODUCTION

Respiratory tract diseases (such as allergic rhinoconjunctivitis (AR) and allergic asthma (AA) induced by outdoor and/or indoor aeroallergens are highly prevalent worldwide and may affect up to 20% of the general population.^1^, ^2^ Although mild disease is rarely bothersome, moderate‐to‐severe symptoms of AR not only impair work/school productivity, sleep quality, and health‐related quality of life^3^, ^4^, ^5^, ^6^, ^7^ but may also accentuate the risk of progression to AA.^8^, ^9^, ^10^ Birch (*Betula*) pollen allergy is the most prevalent tree pollen allergy in temperate regions, such as northern and central Europe.^11^ The duration and intensity of birch pollen exposure allergy are increasing as a result of climate change.^12^ Cross‐reactivity with homologous pollen allergens from other trees (notably Alder, beech, chestnut, hazel, hornbeam, and oak) is also observed.^13^ Lastly, cross‐reactivity between common food allergens (mainly in apples, cherries, peaches, plums, and hazelnuts) and the Bet v 1 birch pollen allergen may trigger oral allergy syndrome in up to 70% of birch‐pollen‐allergic patients.^14^, ^15^ The symptoms of birch pollen AR can be relieved (at least temporarily) with symptomatic medications, including H1 antihistamines, leukotriene receptor antagonists, and corticosteroids.^16^, ^17^, ^18^ However, these medications may lack efficacy or be poorly tolerated. The only currently available, systemic, disease‐modifying treatment for allergic respiratory diseases is allergen immunotherapy (AIT).^19^, ^20^

By acting directly on the immune system, AIT induces tolerance to the disease‐inducing allergen.^21^, ^22^, ^23^ After over a century of clinical use and the development of modern pharmaceutical formulations over the last decades, both subcutaneous allergen immunotherapy (SCIT) or sublingual allergen immunotherapy (SLIT) have proven their efficacy and safety in the treatment of AR and/or AA induced by a range of allergens, including pollens.^18^, ^23^, ^24^, ^25^, ^26^, ^27^, ^28^, ^29^, ^30^, ^31^, ^32^, ^33^ A variety of pharmaceutical formulations of birch pollen extracts have been marketed for SCIT or SLIT in patients with moderate‐to‐severe birch pollen allergy.^34^, ^35^, ^36^ One such formulation is depigmented polymerized birch SCIT, which has been marketed as a named patient preparation at doses of up to 1000 DPP/mL (corresponding to ∼7 μg Bet v 1/mL prior to depigmentation) in Spain and Germany.^34^, ^37^, ^38^ The results of a dose‐ranging study (EudraCT 2008‐008448‐26, NCT01144429) of 100, 1000, 5000 and 10,000 DPP/mL formulations suggested that the 5000 DPP/mL dose level had an even more favourable efficacy and a comparable safety profile to the 1000 DPP/mL dose level.^39^ The present study constituted part of the Phase III clinical development of this 5000 DPP/mL formulation (EudraCT 2012‐000414‐11).

As mentioned above, the mode of action of AIT is based on long‐term tolerance of disease‐inducing allergens. Currently, regulatory authorities take account of the time scale of efficacy for AIT products. The European Medicines Agency (EMA) recommends that AIT preparations have (i) short‐term efficacy (i.e. in the first season or year of treatment), (ii) sustained efficacy (i.e. the maintenance of efficacy for two to three seasons or years), and (iii) long‐term efficacy (following the withdrawal of treatment).^40^ Most guidelines recommend a 3‐year course of AIT, and this duration is supported by data from the literature.^27^ Hence, the overall goal of this multinational, multicentre, double‐blind, placebo‐controlled (DBPC) randomized clinical trial with two parallel groups was to demonstrate the clinical efficacy of a birch 5000 DPP/mL SCIT preparation (vs. placebo) during 3 years of treatment and two post‐treatment years^25^, ^26^, ^41^ in adults and adolescents with birch pollen AR (with or without intermittent asthma).

The primary objective of the present study was therefore to test the superiority of active treatment with regard to a combined symptom and medication score (CSMS). The secondary objectives were to establish the active treatment's efficacy with regard to other endpoints (including symptom scores, a rescue medication (RM) score, disease‐specific quality of life, and immunological parameters) and to assess local and systemic safety and tolerability.

## 2 METHODS

### 2.1 Trial design

We performed a Phase III multinational, multicentre, DBPC randomized clinical trial with two parallel groups. The study centres were located in the Czech Republic, Finland, Germany, Latvia, Lithuania, Poland, and Russia. There were two recruitment periods: the first ran from September 2012 to January 2013, and the second started after the end of the 2013 birch pollen season (August 2013) and continued until January 2014. After a screening phase, eligible patients were randomized to active treatment (see below) or placebo. The study comprised an 8‐week screening phase, a 1‐day initial build‐up phase, a blinded 3‐year maintenance treatment phase (including three birch pollen seasons, with treatment administered monthly) and a 2‐year blinded, treatment‐free follow‐up phase (including 2 birch pollen seasons). The total study duration per patient was approximately 5 years.

Start‐ and end‐dates of local pollen seasons were determined by local pollen exposure reports for each study centre. The start date of relevant birch pollen exposure was defined as the first of three consecutive days with a pollen count ≥50 grains/m^3^/24 h.^38^ The end date of the birch pollen season was defined as the day one week after the last day of the year with ≥50 pollen grains/m^3^/24 h.

### 2.2 Trial population

The study's main inclusion criteria were as follows: (i) age from 12 to 70 at the time of screening; (ii) a physician‐documented history of at least 2 years of treated seasonal AR with or without intermittent asthma; (iii) self‐rated, moderate‐to‐severe symptoms in at least the two previous birch pollen seasons; (iv) peak expiratory flow ≥80% predicted; (v) IgE‐mediated sensitization to birch pollen allergen, as defined by a suggestive medical history, serum specific IgEs (sIgEs) to birch pollen and a positive (wheal diameter ≥3 mm) skin prick test (SPT) to birch pollen extract at the screening visit or during the previous month; (vi) Internet access (for daily completion of an electronic patient diary), and (vii) prior written informed consent to participation in the study.

The main exclusion criteria were (i) a history of potentially confounding symptoms triggered by allergens other than birch pollen (grass pollen, weed pollen, house dust mites, and cat or dog dander) based on specific IgEs and SPT, (ii) moderate or severe persistent asthma (Global Initiative for Asthma [GINA] grade 3 or 4^42^), (iii) mild persistent asthma (GINA 2) but that necessitated treatment with inhaled glucocorticoids at a daily dose level of >400 μg budesonide dose equivalent, (iv) past or present severe atopic dermatitis, (v) AIT with any allergen in the previous 6 months or with birch pollen AIT in the previous 5 years, (vi) a probable change in the place of residence during and between birch pollen seasons, and (vii) standard contraindications to AIT, according to recent guidelines.^28^, ^43^, ^44^

### 2.3 Randomization

Included patients were assigned a unique identifier (a 3‐digit site number, followed by an increasing 3‐digit patient number) before randomization 2:1 to active treatment or placebo. The randomization schedule was generated by a clinical research organization using dedicated software and was not known to the investigators or other study personnel. Copies of the randomization schedule were provided to (i) LETI Pharma (Tres Cantos, Spain) for supply of the active treatment, and (ii) a clinical trial supplies and services provider responsible for the distribution of the active treatment and non‐investigational medical products and for ad hoc substitution of active treatment kits in the event of damage (e.g. freezing). For emergency un‐blinding, the investigational sites received sealed code cards corresponding to the active treatment kit numbers they had received for their patients.

### 2.4 Study treatments

The active treatment for SCIT was Birch 5000 DPP/mL (LETI Pharma), a depigmented polymerized allergen extract adsorbed onto aluminium hydroxide and suspended in 0.9% NaCl with 0.5% phenol. The DPP unit corresponds to the depigmentation and polymerization of 1 histamine equivalent (in an SPT) in the allergenic extract. The histamine equivalent unit was 10 mg/ml. The 5000 DPP/mL dose corresponds to a titre of ∼35 μg Bet v1/mL prior to depigmentation. To maintain blinding, the placebo SCIT formulation had the same appearance as the active treatment. The treatment regimen comprised a rush build‐up phase (two separate injections—0.2 and 0.3 mL—administered 30 min apart on day 1) and a maintenance phase (one 0.5 mL injection per visit at 4‐ to 6‐week intervals for up to 36 months, giving a total of up to 29 maintenance injections).

### 2.5 Endpoints and assessments

The initially specified primary efficacy variable was a CSMS that ranged from 0 (best possible) to 36 (worst possible) per day (daily symptom score (dSS): 0–18; daily RMS (dRMS): 0–18). Calculation of this CSMS (referred to hereafter as “CSMS_0‐36_”) is described in more detail in the Supplementary Material. During the course of the study, the European Academy of Allergy and Clinical Immunology (EAACI) recommended an adaptation of the CSMS, which we decided to implement as a *post hoc* primary efficacy endpoint. This score (referred hereafter as the “CSMS_EAACI_”) took account of four nasal symptoms (nasal pruritus, rhinorrhoea, congestion, and sneezing) and two ocular symptoms (ocular pruritus/grittiness/redness, and tearing) over the pollen season. The dSS is divided by the number of symptoms scored (to give a value from 0 to 3) and is added to the corresponding dRMS (scored from 0 to 3; see the Supplementary Material), giving CSMS = dSS + dRMS (range: 0–6). The median score was calculated as the median area under the curve for linearly interpolated scores recorded in the patient's e‐diary between first and last diary entries per local pollen season and was standardized against the duration of the pollen season.

Another CSMS (“CSMS_lung_”) that additionally included four pulmonary symptoms (wheezing, coughing, breathlessness, and tightness of the chest) and the corresponding RMs taken for these symptoms was evaluated as a secondary efficacy endpoint. The other secondary endpoints are listed in Supplementary Table 2. For the analysis of safety, adverse events (AEs) were recorded, classified as serious or not serious (as defined in Supplementary Table 2), and graded by system organ class and preferred term according to the Medical Dictionary for Regulatory Activities (MedDRA, www.meddra.org). Lastly, the intensity of each AE was rated as mild (tolerable), moderate (bothersome, interference with usual activities) or severe (inability to perform usual activities).

In order to investigate potential confounder variables and their possible impact on the treatment effect of birch 5000 DPP/mL versus placebo, an explorative post‐hoc confounder analysis was performed.

### 2.6 Sample size calculation and statistical analysis

The sample size calculation was based on the sponsor's previous clinical studies in this indication, with the following assumptions: an expected mean CSMS_0‐36_ in the placebo group of 8.5 in pollen season 1 and 8.0 in the following treatment years, an expected mean CSMS_0‐36_ in the active treatment group of 7.0 in pollen season 1 and 6.0 in the following treatment years; a typical standard deviation (SD) of 5, a participant drop‐out rate of 10% per study year (i.e. 50% after 5 years), a normal data distribution, a family‐wise error rate of 0.025 (one‐sided), and a relative effect size of 0.4.

A futility analysis (to potentially provide evidence of non‐inferiority vs. placebo or to stop the study for futility) was scheduled at the end of year 1. Interim analyses (to provide evidence of efficacy vs. placebo) were scheduled at the end of year 2 and the end of year 3. Given that the second interim analysis did not show superiority of the active treatment over placebo for the full analysis set (FAS), the study continued with monosensitized, mono‐allergic patients only (see the Results section) according to SPT reaction. This subset comprised participants who had a positive SPT reaction only against birch pollen allergens at the screening visit. These patients were analyzed on a post hoc basis.

Continuous variables were expressed as the mean (SD) or mean (range) or, when not normally distributed, the median [interquartile range (IQR)] or median (range). Categorical variables were expressed as the frequency (percentage). All statistical analyses were performed with SAS^®^ software (version 9.4, SAS Institute Inc., Cary, NC).

### 2.7 Ethics

All participants aged 18 or over gave their written consent to participation, after having been informed of the study's objectives and procedures. Participants aged 12 to 17 gave their assent to participation, and the participants' parents or legal representatives gave their written consent. The study was approved by the appropriate independent ethics committees in each country and was registered in the European Clinical Trials Database (EudraCT 2012‐000414‐11).

## 3 RESULTS

### 3.1 Study participants

**FIGURE 1:**
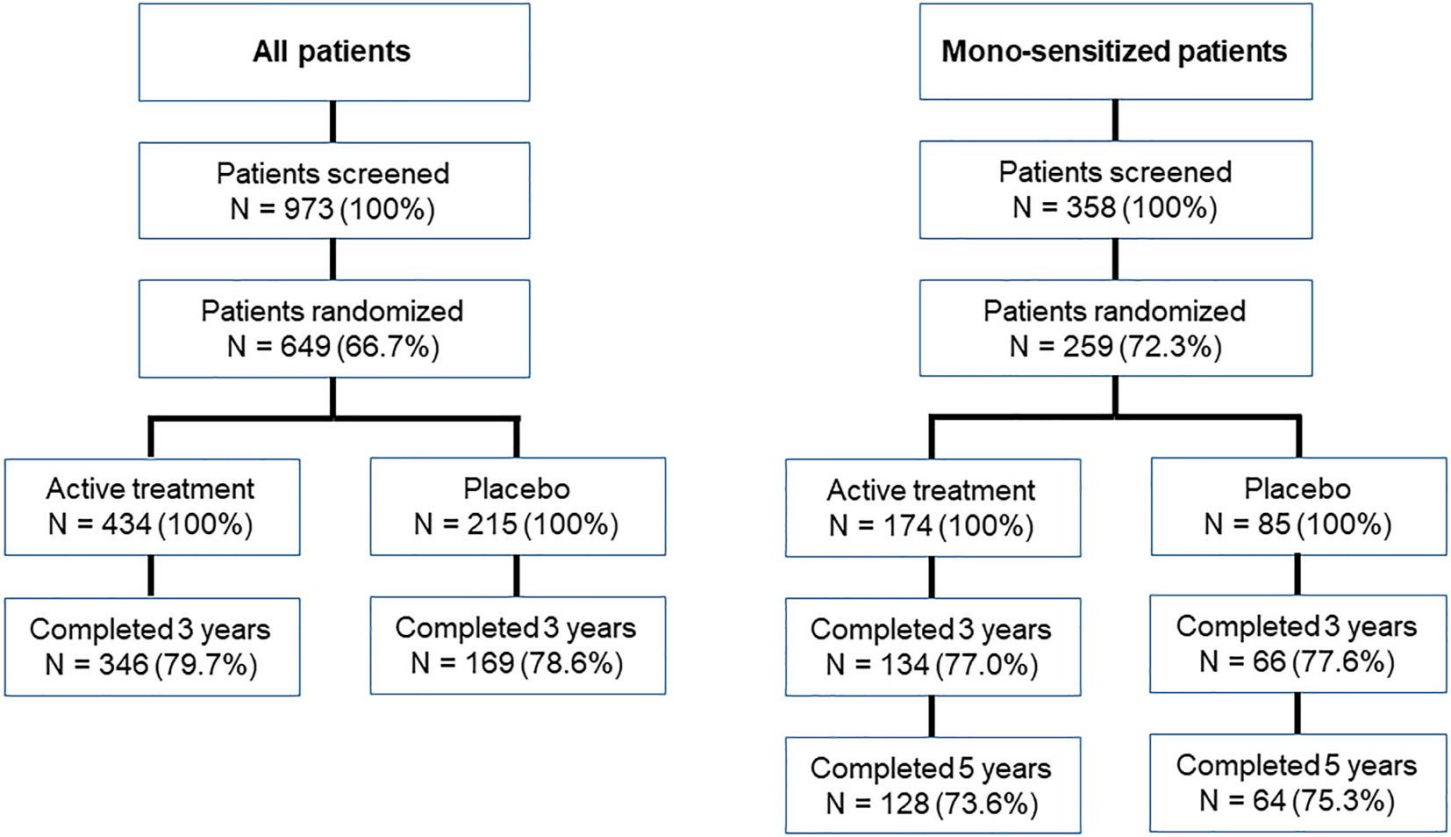
Patient disposition

Sixty‐six sites (7 in the Czech Republic, 4 in Finland, 32 in Germany, 2 in Latvia, 4 in Lithuania, 12 in Poland, and 5 in Russia) enrolled and treated patients. A total of 973 participants were screened, 649 were randomized (also corresponding to the safety set), 515 completed 3 years of the study, and 200 monosensitized participants completed 5 years. The first inclusion visit took place on September 17th, 2012, and the last patient visit took place on July 30th, 2018 (Figure 1). 41 patients randomized were excluded due to screening failure, therefore, the full analysis set (FAS) comprised 608 participants (406 in the active treatment group and 202 in the placebo group; 48 adolescents (7.9%) and 560 adults (92.1%)). The mean age was 37.6 and there was slight female predominance (*n* = 333, 54.8%). The great majority of the patients did not consume alcohol on a daily basis and had never smoked. The demographic and clinical characteristics of the active treatment and placebo groups were similar, as were those of the overall FAS and the subset of monosensitized patients (with the exception of sensitization status) (Table 1). Around one third of the participants in each treatment group had a history of asthma (active treatment: 29.1%; placebo: 31.2%). The per protocol set comprised 573 participants in year 1 (387 and 186 in the active treatment and placebo groups, respectively), 534 in year 2 (363 and 171), and 497 in year 3 (336 and 161). Following the results of the second interim analysis, the study continued with the subset of 240 monosensitized participants. The level of treatment compliance was high (96.4% overall, 95.7% in the active treatment group, and 96.2% in the placebo group).

**TABLE 1:** Demographic characteristics of the study population polysensitized versus monosensitized patients

Parameter	Category/statistic	Polysensitized active treatment (*N* = 245)	Polysensitized placebo (*N* = 123)	Polysensitized total (*N* = 368)	Monosensitized active treatment (*N* = 161)	Monosensitized placebo (*N* = 79)	Monosensitized total (*N* = 240)
Age (years)	Mean (SD)	37.1 (13.85)	35.6 (13.06)	36.6 (13.59)	38.4 (12.71)	40.8 (12.99)	39.2 (12.82)
	Median (range)	37.0 (12–70)	34.0 (12–67)	36.0 (12–70)	38.0 (12–70)	41.0 (13–69)	39.0 (12–70)
Sex	Male	117 (4.78)	55 (44.7)	172 (46.7)	69 (42.9)	34 (43.0)	103 (42.9)
	Female	128 (52.2)	68 (55.3)	196 (53.3)	92 (57.1)	45 (57.0)	137 (57.1)
Ethnicity	White	244 (99.6)	122 (99.2)	366 (99.5)	161 (100)	78 (98.7)	239 (99.6)
	Other	1 (0.4)	1 (0.8)	2 (0.6)	0	1 (1.3)	1 (0.4)
Weight (kg)	Mean (SD)	74.8 (16.21)	73.0 (15.97)	74.2 (16.13)	73.5 (17.81)	72.7 (13.75)	73.3 (16.56)
	Median (range)	74.0 (39–130)	70.0 (39–125)	73.0 (39–130)	70.0 (42–145)	70.0 (39–102)	70.0 (39–145)
BMI (Kg/m^2^)	Mean (SD)	25.1 (4.42)	24.7 (4.01)	24.9 (4.29)	24.7 (4.6)	25.4 (4.3)	24.9 (4.5)
	Median (range)	24.4 (15–41)	24.2 (17–36)	24.4 (15–41)	24.5 (17–42)	25.1 (15–37)	24.5 (15–42)

Abbreviations: BMI, body mass index; FAS, full analysis set; N, number of patients in the treatment group; *n*, number of patients with data; %, percentage of N; SD, standard deviation.

### 3.2 Pollen seasons

**FIGURE 2:**
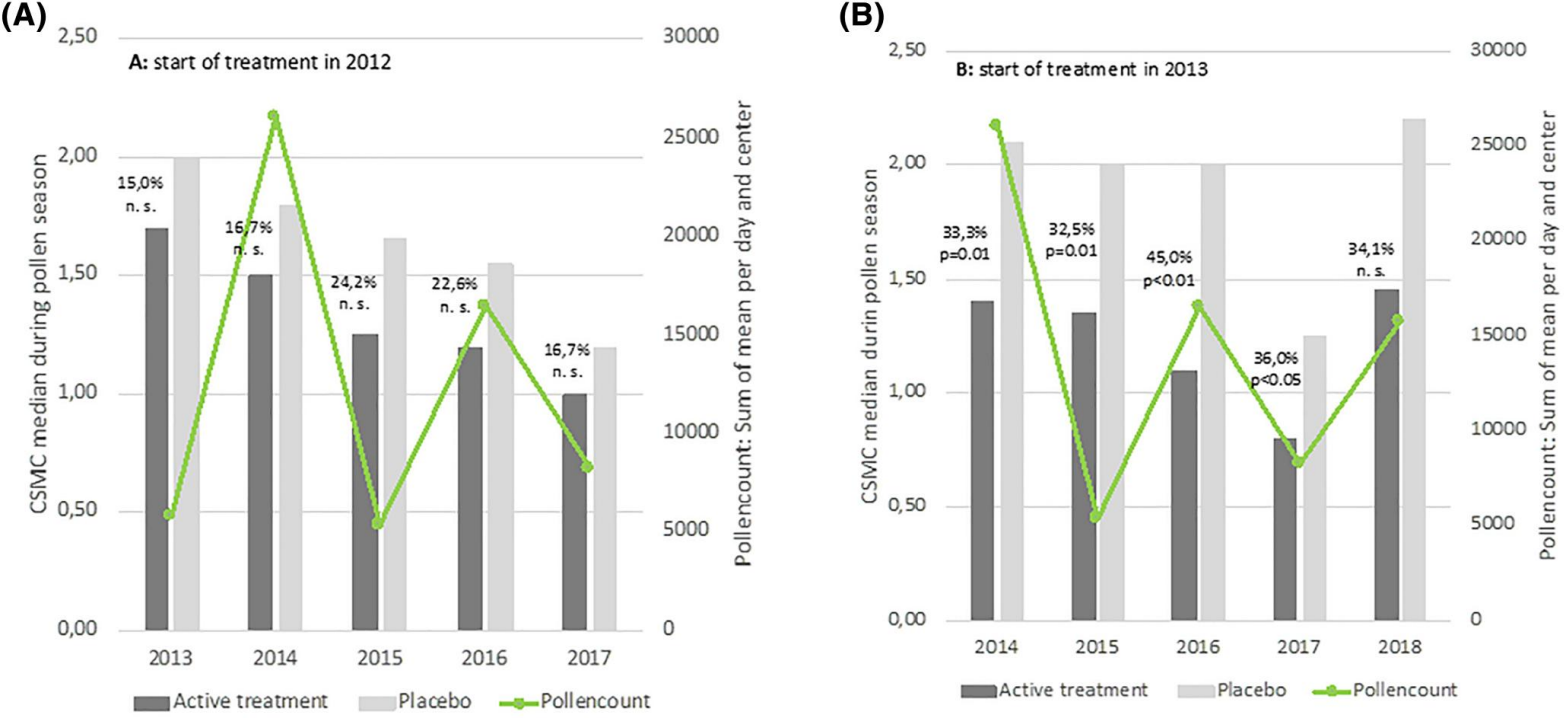
CSMS_(EAACI)_ in the active treatment group compared to the placebo group in each pollen season and faced to the pollen counts in each year, in monosensitized patients. (A) Patients started treatment in 2012. (B) Patients started treatment in 2013

The mean pollen count differed significantly from centre to centre and from year to year. The pollen count was low in 2013 (the first pollen season observed) and four times higher in 2014. This trend continued: the pollen counts were low in 2015 and 2017 and two to three times higher in 2016 and 2018 (Figure 2).

### 3.3 Primary endpoint

The post‐hoc analysis of the primary endpoint (CSMS_EAACI_) (which was changed from CMCS 0–36 during the study as already mentioned above) for the study population as a whole did not show statistically significant differences between active treatment and placebo groups for the pollen seasons in years 1, 2 and 3 (data not shown, *p*‐values >0.05). However, the CSMS_EAACI_ for rhinoconjunctivitis symptoms in year 3 was significantly lower among monosensitized patients having received the active treatment than in those having received the placebo (1.2 (0.70–1.90) versus 1.75 (1.10–2.30), respectively; *p* = 0.0020; Table 2). For year 5 (i.e. after two AIT‐free years), the median [IQR] CSMS_EAACI_ was still lower among monosensitized patients having received the active treatment, and the difference versus placebo was significant (1.10 (0.60–1.90) versus 1.60 (0.80–2.40), respectively; *p* = 0.013; Table 2). Similarly, a statistically significant active treatment versus placebo difference was also found for the median CSMS_lung_ in year 3 (*p* = 0.0057) and year 5 (*p* = 0.0489) (Table 2). Furthermore, the mean (SD) CSMS_0‐36_ was significantly lower in mono‐allergic birch‐SCIT‐treated patients in year 2 (7.37 (4.131), versus 8.93 (5.317) for placebo; *p* = 0.0389) and in year 3 (6.49 (4.166), versus 8.21 (4.538) for placebo; *p* = 0.0040) (Supplementary Table 5).

**TABLE 2:** The median CSMS_EAACI_ and CSMS_lung_ at the end of the treatment period (Year 3) and the end of the treatment‐free follow‐up period (Year 5) in the monosensitized participants, by treatment group

Score	Statistic	Active treatment	Placebo	[Placebo ‐ active treatment]		
					Difference for the median	
				Mean difference	[95%CI**]	*p*‐value*
Year 3	*n*	137	66		[0.200, 0.700]	0.0020
CSMS_EAACI_	Mean (SD)	1.36 (0.882)	1.74 (0.868)	−21.8%		
	Median [IQR]	1.20 (0.70–1.90)	1.75 (1.10–2.30)			
Year 3	*n*	137	66		[0.600, 3.700]	0.0057
CSMS_lung_	Mean (SD)	7.68 (5.629)	9.64 (5.520)	−20.3%		
	Median [IQR]	6.50 (4.00–9.50)	8.50 (5.90–13.30)			
Year 5	*n*	124	63		[0.100, 0.700]	0.0130
CSMS_EAACI_	Mean (SD)	1.30 (0.871)	1.68 (1.012)	−22.6%		
	Median [IQR]	1.10 (0.60–1.90)	1.60 (0.80–2.40)			
Year 5	*n*	124	63		[0.000, 3.200]	0.0489
CSMS_lung_	Mean (SD)	7.22 (5.063)	9.13 (6.346)	−20.9%		
	Median [IQR]	5.85 (3.50–10.00)	7.70 (4.40–13.30)			

Abbreviations: FAS, full analysis set; IQR, interquartile range; *n*, number of patients with data; SD, standard deviation.

*The Hodges‐Lehmann two‐sided 95% confidence interval for the median difference.

**In a two‐tailed Wilcoxon‐Mann‐Whitney test.

When considering the monosensitized patients from the two recruitment periods separately (Supplementary Tables 3 and 4), those who started treatment in 2012 experienced a first pollen season with a comparatively low pollen load (2013: 5895 grains/m³/24 h). In contrast, the patients who started treatment in 2013 experienced a first pollen season with a four‐fold higher pollen load (2014: 26,038 grains/m³/24 h). The treatment effect was clearly greater among the patients exposed to a high pollen load during the first season. In the latter group, the active versus placebo difference in the CSMS_EAACI_ ranged from 32.5% in year 2%–45.0% in year 3 (Figure 2 and Supplementary Table 4). All the year‐wise differences in the CSMS_EAACI_ (except that in year 5) were statistically significant. In the subset who started treatment in 2012 and experienced a low pollen load during the first season, the active versus placebo difference in the CSMS_EAACI_ ranged from 15.0% in year 3%–24.2% in year 3 (Figure 2 and Supplementary Table 3). None of these year‐wise differences were statistically significance during the entire observation period.

### 3.4 Secondary endpoints (in monosensitized patients)

An analysis of the median total symptom score demonstrated a statistically significant difference in favour of active treatment (23.8% lower than placebo) at the end of the treatment phase only (year 3, *p* = 0.0342). The median RMS was low in both treatment groups, indicating that RMs were rarely used by the study patients. However, there were statistically significant differences in favour of the active treatment in years 3 and 5 (62.5% lower than placebo, *p* = 0.00013, and 57.1% lower than placebo, *p* = 0.0032, respectively; Table 3). Like‐wise, the median integrated RMS for asthmatic patients was lower in the active treatment group than in the placebo group in year 3 (*p* = 0.0405) and year 5 (*p* = 0.0635). The mean number of well days increased faster over time in the active treatment group than in the placebo group, although the differences were not statistically significant (Table 3). The mean number of hell days decreased over time in the active treatment group and was stable over time in the placebo group; at the end of the treatment period, the difference was statistically significant (*p* = 0.0019).

**TABLE 3:** Selected secondary efficacy endpoints and immunology parameters in monosensitized participants

Time	Statistics	Active treatment	Placebo	Difference [placebo ‐ active treatment]	
				95% CI**	*p*‐value*
Symptom score (0–3)					
Year 3	*n*	134	66	[0.000, 0.300]	0.0342
	Mean (SD)	0.87 (0.496)	1.01 (0.513)		
	Median (IQR)	0.80 (0.50–1.20)	1.05 (0.60–1.40)		
Year 5	*n*	124	63	[0.000, 0.300]	0.1548
	Mean (SD)	0.84 (0.499)	0.98 (1.622)		
	Median (IQR)	0.80 (0.50–1.10)	0.90 (0.60–1.50)		
RMS (0–3)					
Year 3	*n*	137	66	[0.100, 0.400]	0.00013
	Mean (SD)	0.49 (0.563)	0.72 (0.556)		
	Median (IQR)	0.30 (0–0.90)	0.80 (0.20–1.00)		
Year 5	*n*	124	63	[0.000, 0.400]	0.0032
	Mean (SD)	0.47 (0.574)	0.70 (0.566)		
	Median (IQR)	0.30 (0–0.80)	0.70 (0.20–1.00)		
Well days					
Year 3	*n*	137	66	[−1.000, 0.000]	0.0649
	Mean (SD)	5.64	3.71		
	Median (IQR)	2.00	0.50		
Year 5	*n*	124	63	[−1.000, 0.000]	0.2132
	Mean (SD)	7.00	5.98		
	Median (IQR)		1.00		
Hell days					
Year 3	*n*	137	66	[0.000, 2.000]	0.0019
	Mean (SD)	2.42	4.86		
	Median (IQR)	2.00	7.00		
Year 5	*n*	124	63	[0.000, 1.000]	0.0836
	Mean (SD)	2.44	4.43		
	Median (IQR)	3.00	6.00		
IgG_4_					
Year 3	*n*	42	18	n.d.	n.d.
	Mean (SD)	139.11 (130.78)	33.37 (51.00)		
	Median (IQR)	70.90 (37.30–199.60)	9.85 (7.90–28.70)		
End of study	*n*	42	18	n.d.	n.d.
	Mean (SD)	44.10 (67.362)	27.69 (41.909)		
	Median (IQR)	19.00 (10.80–54.80)	9.60 (5.50–38.00)		
IgE					
Year 3	*n*	120	54	n.d.	n.d.
	Mean (SD)	27.88 (23.659)	33.97 (30.139)		
	Median (IQR)	20.30 (8.71–40.10)	22.20 (5.18–60.90)		
End of study	*n*	121	59	n.d.	n.d.
	Mean (SD)	17.31 (17.554)	21.30 (21.811)		
	Median (IQR)	11.30 (6.08–23.40)	12.70 (4.70–28.90)		

Abbreviations: FAS, full analysis set; IQR, interquartile range: *n*, number of patients with data; SD, standard deviation.

*Wilcoxon‐Mann‐Whitney two‐sided *p*‐value.

**Hodges‐Lehmann two‐sided 95% confidence interval of the median difference.

**FIGURE 3:**
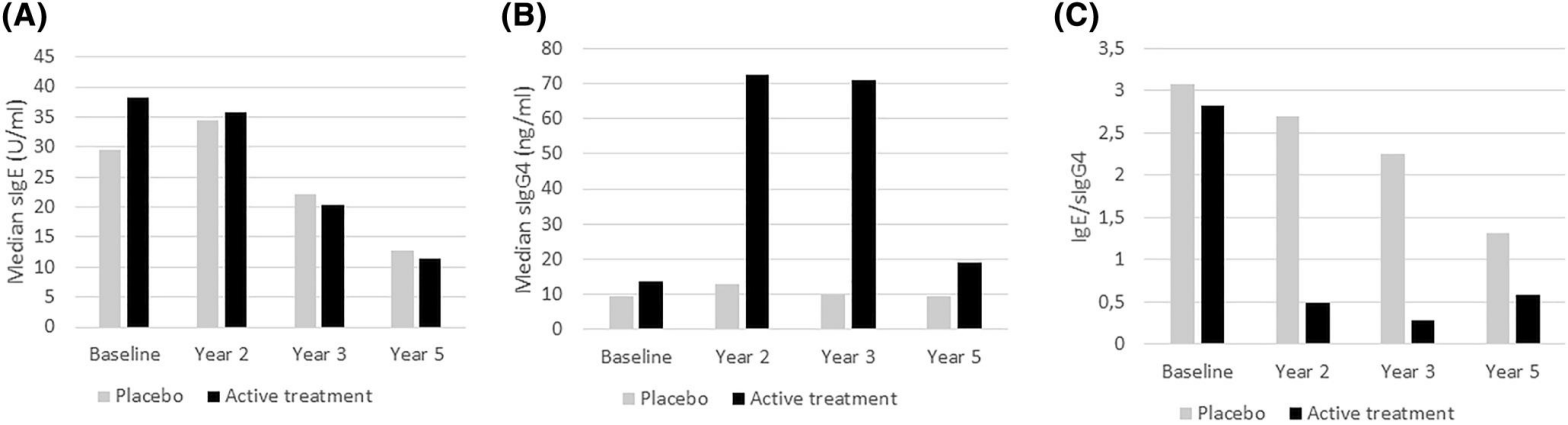
Immunological parameters from mono‐sensitized patients, FAS: (A) sIgE (U/l); (B) sIgG4 (ng/ml) – German sites; (C) sIgE/sIgG4

With regard to immunological variables for mono‐sensitized patients, the mean titre of birch‐pollen‐specific IgE was lower in the active treatment group than in the placebo group in year 3 and year 5, while the mean titre of birch‐pollen‐specific IgG4 was higher in the active treatment group than in the placebo group in year 3 and year 5 (Table 3 and Figure 3).

The post‐hoc confounder analysis of the data for monosensitized patients revealed a trend towards lower efficacy in patients with a higher body mass index (BMI). In year 2, the *p*‐value was <0.05 for the CSMS_EAACI_ × BMI interaction (*p* = 0.043). In year 4 and year 5, the *p*‐value was <0.05 for the CSMS_EAACI_ × treatment interaction (*p* = 0.017 and *p* = 0.043, respectively). Confounding effects of the SPT wheal diameters from ≤13 mm up to ≤24 mm were indicated in year 3 and year 4. Furthermore, an active versus placebo difference in the mean CSMS_EAACI_ x SPT wheal diameter from ≤13 mm up to ≤24 mm was seen throughout the maintenance‐treatment phase (years 1–3) and in year 4 (post‐treatment). Confounding effects on the CSMS_EAACI_ were also seen with the patient's perception of disease activity throughout the study (i.e. including years 4 and 5).

### 3.5 Safety

There was no difference in the incidence of systemic reactions (SRs) between the active treatment and placebo groups during the 3‐year treatment period. Overall, 24.7% of the patients in the active treatment group and 26.0% of the patients in the placebo group experienced at least one SR during the 5 years of the study. Only one Grade 3 SR in the active treatment group and two in the placebo group were reported. There were no grade 4 SRs. The incidence of local reactions in the active treatment group was slightly higher (40.3% of the participants) than in the placebo group (29.8%) (Supplementary material Table 6). The overall frequency was highest in year 1 and tended to decrease over time. In the active treatment group, around 42% of all treatment‐emergent adverse events (TEAEs) were judged to be related to treatment. The frequency of serious related TEAEs was 9.0% (*n* = 39) in the active treatment group and 9.3% (*n* = 20) in the placebo group. The most frequently reported serious TEAEs were osteoarthritis, appendicitis, and breast cancer (each in 3 patients) but these were not considered to be related to the study treatment. No treatment‐related serious TEAEs or TEAEs leading to death were reported during the study. Study discontinuation was prompted by a TEAE in 17 (3.9%) patients in the active treatment group and 6 (2.8%) patients in the placebo group (Supplementary material Table 6). The frequency of TEAEs was similar across all patient sets.

The overall assessment of the active treatment's tolerability was “excellent” or “good” in 91.6% of patients (according to the investigator) or in 87% (according to the patients themselves).

With regard to patients with asthma, the vast majority in both treatment groups reported no changes or even an improvement in their asthma status during the study.

## 4 DISCUSSION

The present study is the first birch pollen SCIT trial to be designed in line with the EMA's guidance on demonstrating short‐term, sustained and long‐term efficacy in the clinical development of AIT products.^40^ The study's objective was thus to highlight the long‐term efficacy (defined by the EMA and endorsed by the EAACI as “significant and clinically relevant efficacy in post treatment years”) of a birch 5000 DPP/mL SCIT preparation versus placebo for the perennial treatment of AR (with or without intermittent asthma) in adult and adolescent patients.^28^, ^40^ In a review published in 2018, Penagos et al. stated that although a number of studies had assessed the long‐term clinical and immunological benefits of SCIT, few had a DBPC design. In fact, Penagos et al identified only three such trials (one on grass pollen SCIT,^26^, ^45^, ^46^ one on ragweed (*Ambrosia*) pollen SCIT,^47^ and one (although only partially double‐blind) on pellitory (*Parietaria judaica* and *Parietaria officinalis*)) pollen SCIT.^48^ Hence, the present trial of birch pollen SCIT now joins this list.

The present study's objective of long‐term efficacy was not met for the study population as a whole: an interim analysis did not yield statistically significant results for the primary endpoint in the whole study population. Hence, the study continued with monosensitized patients only. In this subgroup, we observed a statistically significant advantage (vs. placebo) in the primary endpoint (CSMS_0‐36_) for birch 5000 DPP/mL in years 2 and 3. The active versus placebo difference in the mean CSMS_0‐36_ after two treatment‐free years (i.e. in year 5) was not significant (*p* = 0.0556), although the median CSMS_EAACI_ was significantly lower in the active treatment group than in the placebo group in year 3 and year 5—suggesting potential long‐term efficacy of birch SCIT in these patients. These results were supported by significant active versus placebo differences in secondary efficacy endpoints (including symptom scores, RMSs, and immune markers) among the subset of monosensitized patients. In the active treatment group, the mean titre of birch‐pollen‐specific IgE was lower (vs. placebo) in years 3 and 5, while the mean titre of birch‐pollen‐specific IgG4 was higher in year 3. These parameters have been demonstrated to mirror AIT's impact on the immune system.^49^

The lack of efficacy in the study population as a whole could not be accounted for by baseline differences between the active treatment and placebo groups, which were small and not statistically significant. Like‐wise, for each birch pollen season, intercenter differences in pollen exposure were not relevant. However, there were differences in the pollen exposure from 1 year to another, and the study's patients were recruited over two pollen seasons. Although the treatment effect appeared to be similar in the group of patients with a high pollen load in the first season and those with a lower pollen load in the first season, we suggest that the strong pollen load might have the primed the immune system towards tolerogenic immune responses, with less severe symptoms and a lower medication need. Interestingly, greater efficacy in the first pollen season led to greater sustained efficacy. Another possible interpretation could be that patients who suffered already during a weak pollen season might feel a stronger treatment effect during the following strong pollen season.

Confounder analyses revealed stronger effect of birch pollen SCIT in mono‐sensitized patients with higher symptom score at onset of the study and larger birch pollen skin prick test wheal diameter. Also a trend could be observed for BMI with a reduced treatment effect in patients with higher BMI.

Our study addresses a longstanding question: the efficacy of specific AIT in polysensitized patients. One hypothesis, which can be drawn from the data is that allergen immunotherapy especially SCIT is only effective if the Th2 dominance is not sustained, or vice versa it is not effective in Th2‐athletes who are polysensitized and may have high total IgE and eosinophils. A major result is that monosensitized patients will profit most, prompting the need for detailed molecular IgE diagnosis.

So far it is unclear, why other studies, in particular SLIT studies which used comparable exclusion criteria for example, history of potentially confounding symptoms triggered by allergens other than birch pollen (grass pollen, weed pollen, house dust mites, and cat or dog dander) reached significant differences even in the group of polysensitized patients.

The study had some limitations—several of which are inherent to placebo‐controlled studies of AIT in which participants are exposed to confounding allergens.^50^ Firstly, and as mentioned above, a high proportion of the patients were mono‐allergic but polysensitized; this might have masked (at least partially) active treatment versus placebo differences. Nevertheless, significant efficacy was seen in the subset of monosensitized, mono‐allergic participants. Secondly, and as also mentioned above, patient recruitment took place over two successive pollen seasons; this introduced a potential source of bias. Thirdly, the recruitment target for adolescent study participants was not met, which prevented a quantitative assessment of the treatment effect in this subgroup.

This was the first long‐term DBPC study of birch pollen SCIT. Large DBPC studies with long‐term follow‐up are complex and expensive to perform; however, they provide a lot of important information on study designs, quality criteria and scores and, ultimately, improve the management of allergic patients in clinical practice.^33^, ^51^ Even when these long‐time studies' primary endpoints are not met (e.g. as in the scientifically informative GAP trial^52^), they provide much additional information via (for example) analyses of secondary endpoints or post‐hoc analyses; their results must be published for ethical and practical reasons.

In conclusion, treatment with Birch 5000 DPP in an indication of birch‐pollen induced AR with or without asthma showed efficacy after 3 years of treatment and long‐term efficacy (compared with placebo) in year 5 in the subset of patients (monosensitized, mono‐allergic individuals). The safety profile was consistent with the literature data accumulated using different doses of birch SCIT formulations.

## AUTHOR CONTRIBUTIONS

All authors contributed substantially to study design, data acquisition, analysis and/or interpretation, and drafting and critical revision of manuscript. All authors approved the version submitted for publication.

## CONFLICT OF INTEREST

Natalija Novak reports grants and personal fees from Abbvie, Allergopharma, Alk Abello, Bencard Allergy Therapeutics, Blueprint, HAL Allergy, Leti Pharma, Leo Pharma, Eli Lilly, Lofarma, Novartis, Pfizer, Regeneron, Sanofi Genzyme, Stallergenes‐Greers, Streamed up, Thermo Fisher. Margitta Worm reports on speaker honoraria and advisory board activity from Alk Abello, Stallergenes, Bencard Allergy Therapeutics, Novartis, Leo Pharma, Thermo Fisher, Aimmune, Abbvie, Eli Lilly, Genzyme, Pfizer, Regeneron and Sanofi Genzyme. She acted as PI in clinical studies with Leti Pharma. Petra Staubach reports grants and personal fees from AbbVie, Allergika, Almirall‐Hermal, Amgen, Beiersdorf, BMS, Boehringer‐Ingelheim, Celgene, CSL‐Behring, Eli‐Lilly, Falk, Galderma, Hexal, Janssen, Klinge, Klosterfrau, LEO‐Pharma, LETI‐Pharma, L'Oreal, Novartis, Octapharma, Pierre Fabre, Pfizer, Pflüger, Pharming, Regeneron, Shire, Takeda, Regeneron, Sanofi‐Genzyme und UCB Pharma. Marek Jutel reports personal fees from Allergopharma, ALK‐Abello, Allergy Therapeutics, Anergis, HAL, LETI and Stallergenes. Angelika Sager is an employee of LETI Pharma GmbH. Oliver Pfaar reports grants and personal fees from ALK‐Abello, Allergopharma, Stallergenes‐Greers, HAL Allergy, Lofarma, Biomay, Circassia, ASIT Biotech Tools SA, LETI Pharma, Meda Pharma/Mylan, Anergis SA, Mobile Chamber Experts, Indoor Biotechnologies, GlaxoSmithKline, Astellas Pharma Global, EUFOREA, Roxall, Novartis, Sanofi‐Aventis and Sanofi‐Genzyme, Med Update Europe GmbH, Immunotek, Regeneron Pharmaceuticals and others. The study was sponsored and funded by LETI Pharma GmbH.

## Supporting information

Supplementary Material
