# Supplementary material for: Subcutaneous birch pollen allergen immunotherapy with a depigmented polymerized extract shows only sustained and long‐term efficacy in a subgroup of monosensitized adults and adolescents with allergic rhinitis
Source: Clin Transl Allergy. 2022 Oct 5;12(10):e12185. doi: 10.1002/clt2.12185 (PMC9533217; doi:10.1002/clt2.12185)
Supplement: Supplementary file 1 — Supplementary Material [file CLT2-12-e12185-s001.docx]

**Supplementary Material**

Calculation of the CSMS

Firstly, for calculation of the symptom score component of the CSMS, the participants graded the severity of their symptoms as follows: 0=absence of symptoms; 1=mild symptoms (present but not bothersome), 2=moderate symptoms (bothersome but tolerable), and 3=severe symptoms (not tolerable; interference with activities of daily living and/or sleeping). Hence, the symptom score component ranged from 0 (no symptoms in any category) to 18 (severe symptoms in all categories).

The authorized rescue medications (RMs) were country-specific and were prescribed to the patients during the birch pollen seasons at the investigator’s discretion. However, levocabastine or azelastine eye drops, levocabastine or azelastine nasal spray, levocetirizine 5 mg tablets, mometasone nasal spray, and methylprednisolone 16 mg tablets were allowed in all countries. The participants were allowed to take the oral corticosteroids in the investigating centre only, and not at home. All RM intake during the pollen season was recorded by the patient in the web-based diary. The study participants were told to take RM in a step-wise manner that can be summarized as step 1=antihistamine eye drops and/or nasal spray and/or oral antihistmine; step 2: add intranasal steroids; step 3: add corticosteroid tablets (51). Each administration of RM (up to a daily limit) was attributed with a score. The main RMs did not include asthma medications. Participants with asthma were authorized to take a short-acting inhaled β2-agonist (Salbutamol) and an inhaled corticosteroid. Further details of the RM scoring are given in Supplementary Table 1. The RMs (excluding RM for asthma) ranged from 0 (no RM taken) to 18 (maximum administration of all categories of RM). Hence, for a given patient on a given day, the CSMS was calculated as the sum of the daily symptom score (dSS) and the daily RM score. It could range from 0 (best possible) to 36 (worse possible). The median CSMS was calculated as the median area under the curve for linearly interpolated CSMS recorded in the patient’s e-diary between first and last diary entries per local pollen season and was standardized against the duration of the pollen season.

| **Rescue medication** | **Formulation** | **Maximum daily dose*** | **Score per application** | **Maximum daily score** |  |
| --- | --- | --- | --- | --- | --- |
| Nasal spray | Levocabastine or azelastine | 2 x 2 puffs/nostril | 1.5 | 3 |  |
| Eye drops | Levocabastine or azelastine | 4 x 1 drop/eye | 1.5 | 6 |  |
| Oral antihistamine | Levocetirizine 5 mg | 1 x 5 mg | 2 | 2 |  |
| Nasal corticosteroid | Mometasone 50 µg/puff | 2 x 200 µg | 2 | 4 |  |
| Oral corticosteroid (nasal symptoms) | Methylprednisolone 16 mg | 1 x 16 mg for 4 days | 3 | 3 |  |
| Oral corticosteroid (lung symptoms)** | Methylprednisolone 16 mg | 1 x 32-40 mg/day | 3.0–4.0 | 3.0–4.0 |  |
| Short-acting β2-agonist** | Salbutamol 100 µg inhaler | 2 x 200 µg=400 µg | 2.0 | 4.0 |  |
| Corticosteroid inhaler* | Budesonide 200 µg | 2 x 200 µg=400 µg | 2.0 | 4.0 |  |
| * According to the corresponding summary of product characteristics. ** Only for participants with asthma. | | | | | |

Supplementary Table 1. Authorized rescue medications and the scoring system

Supplementary Table 2. Secondary and safety endpoints

| Endpoint | Description |
| --- | --- |
| *Secondary endpoints* | |
| Rhinoconjunctivitis symptom score | Nasal pruritus, rhinorrhoea, congestion, sneezing, ocular pruritus/grittiness/redness, and tearing; each rated from 0 to 3. Range: 0 (best possible) to 18 (worst possible) |
| Lung symptom score | Wheezing, coughing, breathlessness, and chest tightness, each rated from 0 to 3. Range: 0 (best possible) to 12 (worst possible) |
| Nose, eye & lung symptom score | Addition of the two above-mentioned scores. Range: 0 (best possible) to 30 (worst possible) |
| RM score | Not including asthma medication |
| RM score + asthma | Including asthma medication |
| CSMS response | A responder analysis based on the proportion of participants with a mean CSMS below or equal to a certain cut-off |
| CSMS_EAACI_ | A CSMS calculated according to the European Academy of Allergology and Clinical Immunology’s guidelines (51) |
| CSMS_lung_ | A CSMS that included 4 lung symptoms and the corresponding RMs, in addition to rhinoconjunctivitis symptoms and RMs |
| RQLQ | Rhinoconjunctivitis Quality of Life Questionnaire (52) |
| AdolRQLQ | Adolescent Rhinoconjunctivitis Quality of Life Questionnaire (53) |
| Immunologic parameters | total serum IgE, serum specific IgE, serum specific IgG_1_, and serum specific IgG_4_ |
| Well days | Days with a rhinoconjunctivitis symptom score ≤2 and no RM use |
| Hell days | Days with a rhinoconjunctivitis symptom score ≥10 and RM use |
| *Safety endpoints* | |
| Adverse events | Any untoward medical occurrence that occurred for the first time or worsened during the course of the study, irrespective of any putative causal relationship with the intake of the study treatment. |
| Serious adverse events | Events that resulted in death, were life-threatening, required or prolonging hospitalization, resulted in persistent or significant disability or incapacity, resulted in a congenital anomaly or birth defect, or constituted an important medical event |
| Vital signs, haematological and clinical chemistry results |  |

Supplementary Table 3. The median CSMS_EAACI_ in monosensitized patients from the first recruitment period (2012)

| **Pollen season** | **Statistic** | **Birch** | **Placebo** | **Pollen count**  **Sum of mean per day and center**  **grains/m³/24 hr** |  |  |
| --- | --- | --- | --- | --- | --- | --- |
|  |  |  |  |  | **Δ %** | **p-value**** |
| Year 1  CSMS (0-6) | n | 92 | 43 | 5,895 | -15.0 | 0.4902 |
|  | Median [IQR] | 1.70  (0-4.40) | 2.00  (0-4.00) |  |  |  |
| Year 2  CSMS (0-6) | n | 84 | 39 | 26,083 | -16.7 | 0.1086 |
|  | Median [IQR] | 1.50  (0-4.90) | 1.80  (0-3.90) |  |  |  |
| Year 3  CSMS (0-6) | n | 74 | 36 | 5,367 | -24.2 | 0.1443 |
|  | Median [IQR] | 1.25  (0-4.20) | 1.65  (0-4.30) |  |  |  |
| Year 4  CSMS (0-6) | n | 69 | 34 | 16,494 | -22.6 | 0.2141 |
|  | Median [IQR] | 1.20  (0.10-4.10) | 1.55  (0-3.90) |  |  |  |
| Year 5  CSMS (0-6) | n | 63 | 33 | 8,357 | -16.7 | 0.0666 |

| **Pollen season** | **Statistic** | **Active treatment** | **Placebo** | **Pollen count**  **Sum of mean per day and center**  **grains/m³/24 hr** |  |  |
| --- | --- | --- | --- | --- | --- | --- |
|  |  |  |  |  | **Δ %** | **p-value**** |
| Year 1  EAACI CSMS | n | 56 | 34 | 26,083 | -33.3 | 0.0175 |
|  | Median [IQR] | 1.40  (0.10-3.10) | 2.10  (0.10-4.10) |  |  |  |
| Year 2  EAACI CSMS | n | 56 | 31 | 5,367 | -32.5 | 0.0172 |
|  | Median [IQR] | 1.35  (0.20-3.00) | 2.00  (0.30-3.80) |  |  |  |
| Year 3  EAACI CSMS | n | 55 | 29 | 16,494 | -45.0 | 0.0091 |
|  | Median [IQR] | 1.10  (0-2.80) | 2.00  (0.30-3.60) |  |  |  |
| Year 4  EAACI CSMS | n | 46 | 28 | 8,357 | -36.0 | 0.0428 |
|  | Median [IQR] | 0.80  (0-2.80) | 1.25  (0.20-3.40) |  |  |  |
| Year 5  EAACI CSMS | n | 54 | 29 | 15,786 | -34.1 | 0.1489 |
|  | Median [IQR] | 1.45  (0.10-3.70) | 2.20  (0.10-3.70) |  |  |  |

Supplementary Table 4 The median CSMS_EAACI_ in monosensitized patients from the second recruitment period (2013)

Supplementary Table 5. The mean CSMS_0-36_ in mono-~~allergic~~ sensitized patients (FAS), by year and by treatment group.

| **Year** | **Statistic** | **Active treatment N=161** | **Placebo**  **N=79** | **Difference in the mean [placebo - active]** | |
| --- | --- | --- | --- | --- | --- |
|  |  |  |  | **[95%CI]*** | **p-value**** |
| Year 1 | n | 148 | 77 | (-0.200, 2.600) | 0.1050 |
|  | Mean (SD) | 7.80 (4.669) | 9.01 (5.222) |  |  |
|  | Median [IQR] | 7.25 (4.35-11.10) | 8.60 (5.50-11.70) |  |  |
| Year 2 | n | 148 | 71 | (0.100, 2.700) | 0.0389 |
|  | Mean (SD) | 7.37 (4.131) | 8.93 (5.317) |  |  |
|  | Median [IQR] | 6.70 (4.50-9.70) | 8.20 (5.00-11.90) |  |  |
| Year 3 | n | 137 | 66 | (0.600, 3.000) | 0.0040 |
|  | Mean (SD) | 6.49 (4.166) | 8.21 (4.538) |  |  |
|  | Median [IQR] | 5.80 (3.80-8.20) | 7.85 (4.70-10.80) |  |  |
| Year 4 | n | 123 | 63 | (-0.200, 2.400) | 0.0974 |
|  | Mean (SD) | 5.82 (3.942) | 7.26 (5.036) |  |  |
|  | Median [IQR] | 5.00 (2.80-8.00) | 6.10 (3.70-10.60) |  |  |
| Year 5 | n | 124 | 63 | (0.000, 2.800) | 0.0556 |
|  | Mean (SD) | 6.27 (4.224) | 7.88 (5.222) |  |  |
|  | Median [IQR] | 5.60 (3.30-8.35) | 6.70 (3.60-10.80) |  |  |

* the Hodges-Lehmann two-sided 95% confidence interval for the median difference.

** in a two-tailed Wilcoxon-Mann-Whitney test.

FAS: full analysis set; IQR: interquartile range; n: number of patients with data; N: number of patients; SD: standard deviation.

Supplementary Table 6: Overview of patients with TEAEs reported during the study (safety set).

| **TEAE** | **Number (%) of patients** | | | | | | |
| --- | --- | --- | --- | --- | --- | --- | --- |
|  | **Active treatment** | | **Placebo** | | **Total** | | |
|  | 1-3 years | 1-5 years | 1-3 years | 1-5 years | 1-3 years | | 1-5 years |
| *Total study population* | | | | | | | |
| N | 434 (100) | - | 215 (100) | - | 649 (100) | | - |
|  | 369 (85.0) | - | 170 (79.1) | - | 539 (83.1) | | - |
| Serious TEAE (n, %) | 39 (9.0) | - | 20 (9.3) | - | 59 (9.1) | | - |
| Treatment-related TEAE (n, %) | 184 (42.4) | - | 69 (32.1) | - | 253 (39.0) | | - |
| TEAE causing discontinuation (n, %) | 17 (3.9) | - | 6 (2.8) | - | 23 (3.5) | | - |
| N with at least 1 SR | 107 (24.7) |  | 56 (26.0) |  | 163 (25.1) | | 259 (100) |
| Any SR Grade 0-2 | 106 (24.4) | 149 (85.6) | 54 (25.2) | 67 (78.8) | 160 (24.6) | | 216 (83.4) |
| Any SR Grad 3 | 1 (0.2) | 14 (8.0) | 2 (0.9) | 7 (8.2) | 3 (0.5) | | 21 (8.1) |
| Any SR Grade 4 | 0 (0.0) | 70 (40.2) | 0 (0.0) | 24 (28.2) | 0 (0.0) | | 0 (0.0) |
| N with at least 1 LR | 175 (40.3) |  | 64 (29.8) |  | 239 (36.8) | |  |
|  |  |  |  |  |  | |  |
| **TEAE** | **Number (%) of patients** | | | | | | |
|  | **Active treatment** | | **Placebo** | | **Total** | | |
|  | 1-3 years | 1-5 years | 1-3 years | 1-5 years | 1-3 years | 1-5 years | |
| *Total study population* | | | | | | | |
| N | 434 (100) | 174 (100)- | 215 (100) | 85 (100) | 649 (100) | 259 (100) | |
| Any TEAE (n, %) | 369 (85.0) | 149 (85.6)- | 170 (79.1) | 67 (78.8) | 539 (83.1) | 216 (83.4) | |
| Serious TEAE (n, %) | 39 (9.0) | 14 (8.0) | 20 (9.3) | 7 (8.2) | 59 (9.1) | 21 (8.1) | |
| Treatment-related TEAE (n, %) | 184 (42.4) | 70 (40.2) | 69 (32.1) | 24 (28.2) | 253 (39.0) | 0 (0,0) | |
| TEAE causing discontinuation (n, %) | 17 (3.9) | 6 (3.4) | 6 (2.8) | 1 (1.2) | 23 (3.5) | 7 (2.7) | |
| N with at least 1 SR | 107 (24.7) | 50 (28.7) | 56 (26.0) | 25 (29.4) | 163 (25.1) | 75 (29.0) | |
| Any SR Grade 0-2 | 106 (24.4) | 40 (23.0) | 54 (25.2) | 22 (25.8) | 160 (24.6) | 62 (24.0) | |
| Any SR Grad 3 | 1 (0.2) | 1 (0.6) | 2 (0.9) | 0 (0.0) | 3 (0.5) | 1 (0.4) | |
| Any SR Grade 4 | 0 (0.0) | 0 (0.0) | 0 (0.0) | 0 (0.0) | 0 (0.0) | 0 (0.0) | |
| N with at least 1 LR | 175 (40.3) | 68 (39.1) | 64 (29.8) | 22 (25.9) | 239 (36.8) | 90 (34.7) | |

N: Number of patients treated; %: percentage based on N; TEAE: treatment-emergent adverse event; SR: systemic reaction; LR: local r Figure

Supplementary Table 6. TEAEs by MedDRA system organ class and preferred term (occurrence in > 5% of monosensitized patients), overall and by year for the 5-year study period

| **Time** | **System organ class**  **(Preferred term)** | **Active treatment** | **Placebo** | **Total** |
| --- | --- | --- | --- | --- |
| Overall | N treated | 174 (100) | 85 (100) | 259 (100) |
| 1-5 years | Any TEAE | 149 (85.6) | 67 (78.8) | 216 (83.4) |
|  | Ear and labyrinth disorders^a^ | 8 (4.6) | 5 (5.9) | 13 (5.0) |
|  | Eye disorders | 19 (10.9) | 11 (12.9) | 30 (11.6) |
|  | Conjunctivitis allergic | 9 (5.2) | 6 (7.1) | 15 (5.8) |
|  | Gastrointestinal disorders^a^ | 21 (12.1) | 9 (10.6) | 30 (11.6) |
|  | General disorders and administration site conditions | 70 (40.2) | 22 (25.9) | 92 (35.5) |
|  | Injection site erythema | 28 (16.1) | 9 (10.6) | 37 (14.3) |
|  | Injection site pruritus | 18 (10.3) | 2 (2.4) | 20 (7.7) |
|  | Injection site reaction | 42 (24.1) | 11 (12.9) | 53 (20.5) |
|  | Injection site swelling | 10 (5.7) | 4 (4.7) | 14 (5.4) |
|  | Immune system disorders^a^ | 12 (6.9) | 2 (2.4) | 14 (5.4) |
|  | Infections and infestations | 105 (60.3) | 46 (54.1) | 151 (58.3) |

|  | Bronchitis | 12 (6.9) | 9 (10.6) | 21 (8.1) |
| --- | --- | --- | --- | --- |
|  | Gastroenteritis | 10 (5.7) | 3 (3.5) | 13 (5.0) |
|  | Influenza | 10 (5.7) | 3 (3.5) | 13 (5.0) |
|  | Nasopharyngitis | 62 (35.6) | 15 (17.6) | 77 (29.7) |
|  | Pharyngitis | 14 (8.0) | 5 (5.9) | 19 (7.3) |
|  | Tonsillitis | 10 (5.7) | 4 (4.7) | 14 (5.4) |
|  | Injury, poisoning and procedural complications^a^ | 17 (9.8) | 3 (3.5) | 20 (7.7) |
|  | Investigations | 40 (23.0) | 26 (30.6) | 66 (25.5) |
|  | FEV decreased | 17 (9.8) | 8 (9.4) | 25 (9.7) |
|  | PEFR decreased | 20 (11.5) | 17 (20.0) | 37 (14.3) |
|  | Musculoskeletal and connective tissue disorders | 29 (16.7) | 10 (11.8) | 39 (15.1) |
|  | Back pain | 14 (8.0) | 4 (4.7) | 18 (6.9) |
|  | Nervous system disorders^a^ | 24 (13.8) | 6 (7.1) | 30 (11.6) |
|  | Respiratory, thoracic and mediastinal disorders | 52 (29.9) | 24 (28.2) | 76 (29.3) |
|  | Asthma | 17 (9.8) | 11 (12.9) | 28 (10.8) |
|  | Cough | 11 (6.3) | 7 (8.2) | 18 (6.9) |
|  | Skin and subcutaneous tissue disorders^a^ | 24 (13.8) | 6 (7.1) | 30 (11.6) |
|  | Surgical and medical procedures^a^ | 10 (5.7) | 6 (7.1) | 16 (6.2) |
|  | Vascular disorders^a^ | 9 (5.2) | 9 (10.6) | 18 (6.9) |
| Year 1 | N treated | 174 (100) | 85 (100) | 259 (100) |
|  | Any TEAE | 115 (66.1) | 48 (56.5) | 163 (62.9) |
|  | Gastrointestinal disorders^a^ | 11 (6.3) | 3 (3.5) | 14 (5.4) |
|  | General disorders and administration site conditions | 63 (36.2) | 21 (24.7) | 84 (32.4) |
|  | Injection site erythema | 24 (13.8) | 9 (10.6) | 33 (12.7) |
|  | Injection site pruritus | 16 (9.2) | 2 (2.4) | 18 (6.9) |
|  | Injection site reaction | 35 (20.1) | 10 (11.8) | 45 (17.4) |
|  | Infections and infestations | 60 (34.5) | 32 (37.6) | 92 (35.5) |
|  | Nasopharyngitis | 30 (17.2) | 8 (9.4) | 38 (14.7) |
|  | Investigations | 20 (11.5) | 8 (9.4) | 28 (10.8) |
|  | PEFR decreased | 9 (5.2) | 6 (7.1) | 15 (5.8) |
|  | Musculoskeletal and connective tissue disorders^a^ | 15 (8.6) | 4 (4.7) | 19 (7.3) |
|  | Nervous system disorders^a^ | 12 (6.9) | 3 (3.5) | 15 (5.8) |
|  | Respiratory, thoracic and mediastinal disorders^a^ | 19 (10.9) | 13 (15.3) | 32 (12.4) |
| Year 2 | N treated | 156 (100) | 74 (100) | 230 (100) |
|  | Any TEAE | 101 (64.7) | 39 (52.7) | 140 (60.9) |
|  | General disorders and administration site conditions | 30 (19.2) | 11 (14.9) | 41 (17.8) |
|  | Injection site reaction | 13 (8.3) | 4 (5.4) | 17 (7.4) |
|  | Infections and infestations | 44 (28.2) | 17 (23.0) | 61 (26.5) |
|  | Nasopharyngitis | 24 (15.4) | 4 (5.4) | 28 (12.2) |
|  | Investigations | 22 (14.1) | 12 (16.2) | 34 (14.8) |
|  | FEV decreased | 9 (5.8) | 5 (6.8) | 14 (6.1) |
|  | PEFR decreased | 11 (7.1) | 6 (8.1) | 17 (7.4) |
|  | Musculoskeletal and connective tissue disorders^a^ | 12 (7.7) | 3 (4.1) | 15 (6.5) |
|  | Respiratory, thoracic and mediastinal disorders^a^ | 13 (8.3) | 3 (4.1) | 16 (7.0) |
| Year 3 | N treated | 142 (100) | 66 (100) | 208 (100) |
|  | Any TEAE | 91 (64.1) | 45 (68.2) | 136 (65.4) |
|  | Eye disorders^a^ | 8 (5.6) | 7 (10.6) | 15 (7.2) |
|  | Gastrointestinal disorders^a^ | 7 (4.9) | 4 (6.1) | 11 (5.3) |
|  | General disorders and administration site conditions | 19 (13.4) | 11 (16.7) | 30 (14.4) |
|  | Injection site reaction | 10 (7.0) | 4 (6.1) | 14 (6.7) |
|  | Infections and infestations | 49 (34.5) | 21 (31.8) | 70 (33.7) |
|  | Nasopharyngitis | 25 (17.6) | 8 (12.1) | 33 (15.9) |
|  | Investigations | 18 (12.7) | 11 (16.7) | 29 (13.9) |
|  | FEV decreased | 8 (5.6) | 4 (6.1) | 12 (5.8) |
|  | PEFR decreased | 10 (7.0) | 7 (10.6) | 17 (8.2) |
|  | Musculoskeletal and connective tissue disorders^a^ | 8 (5.6) | 5 (7.6) | 13 (6.3) |
|  | Nervous system disorders^a^ | 9 (6.3) | 3 (4.5) | 12 (5.8) |
|  | Respiratory, thoracic and mediastinal disorders^a^ | 23 (16.2) | 10 (15.2) | 33 (15.9) |
|  | Skin and subcutaneous tissue disorders^a^ | 10 (7.0) | 4 (6.1) | 14 (6.7) |
| Year 4 | N treated | 138 (100) | 66 (100) | 204 (100) |
|  | Any TEAE | 41 (29.7) | 20 (30.3) | 61 (29.9) |
|  | Infections and infestations | 22 (15.9) | 7 (10.6) | 29 (14.2) |
|  | Nasopharyngitis | 9 (6.5) | 2 (3.0) | 11 (5.4) |
|  | Investigations^a^ | 8 (5.8) | 7 (10.6) | 15 (7.4) |
| Year 5^a^ | N treated | 132 (100) | 64 (100) | 196 (100) |
|  | Any TEAE | 18 (13.6) | 7 (10.9) | 25 (12.8) |

^a^ Occurrence of TEAEs in SOCs or PTs within SOCs affected ≤5% of patients.

FEV: forced expiratory volume; MedDRA: Medical Dictionary for Regulatory Activities; N: number of patients treated; %: percentage based on N; PEFR: peak expiratory flow rate; PT: preferred term; SOC: system organ class; TEAE: treatment-emergent adverse event
